# Effect of a nurse-led multidisciplinary team model on chemotherapy-induced nausea and vomiting in lung cancer patients

**DOI:** 10.3389/fonc.2026.1828714

**Published:** 2026-05-08

**Authors:** Wei Liu, Qian Peng, Xiuni Gan

**Affiliations:** 1 Clinical Center For Tumer Therapy, The Second Affiliated Hospital of Chongqing Medical University, ChongQing, China; 2 Nursing department, The Second Affiliated Hospital of Chongqing Medical University, ChongQing, China

**Keywords:** chemotherapy-induced nausea and vomiting, lung cancer, multidisciplinary team, nurse-led, quality of life

## Abstract

**Objective:**

To explore the effect of a nurse-led multidisciplinary team (MDT) model on chemotherapy-induced nausea and vomiting (CINV) in lung cancer patients.

**Methods:**

A total of 126 non-small cell lung cancer patients undergoing platinum-based chemotherapy were divided into a control group (routine care, n=63) and an experimental group (nurse-led MDT intervention, n=63). The primary outcome was the incidence of grade ≥II CINV. Secondary outcomes included scores on the Functional Living Index-Emesis (FLIE), patient cognition of CINV, and satisfaction with CINV management.

**Results:**

The experimental group showed significantly lower frequency and severity of nausea and vomiting at day 5 of chemotherapy cycle 2 (P<0.05). FLIE scores, patient cognition, and satisfaction were all significantly higher in the experimental group (all P<0.001). No severe adverse events were reported during follow-up.

**Conclusion:**

The nurse-led MDT model effectively reduces the severity of CINV, improves quality of life and patient satisfaction, and enhances the standardized management of CINV.

## 1 Introduction

Primary lung cancer is most common malignant tumor in China. According to the latest national mortality surveillance data, lung cancer has become the leading cause of cancer−related death in China, imposing a heavy burden on public health and quality of life (1). Histopathologically, lung cancer is classified into non−small cell lung cancer (NSCLC) and small cell lung cancer (SCLC). NSCLC accounts for more than 80% of cases, mainly including adenocarcinoma and squamous cell carcinoma, while SCLC accounts for the remainder. At present, the major treatments for lung cancer include surgical resection, systemic chemotherapy, and local radiotherapy. Among these, chemotherapy is one of the most important pharmacological treatments, including neoadjuvant, adjuvant, and palliative chemotherapy. Although chemotherapy prolongs patient survival, it also induces a series of adverse reactions. Chemotherapy−induced nausea and vomiting (CINV) is one of the most distressing complications for patients undergoing chemotherapy (2). To maintain patients’ physical and mental status, ensure treatment adherence, and support smooth completion of chemotherapy, effective prevention and management of CINV have become indispensable components of comprehensive lung cancer care (3).

CINV refers to nausea and vomiting directly caused or triggered by chemotherapeutic agents. It is a distressing and debilitating adverse reaction of cancer treatment, with a considerable incidence affecting a substantial proportion of patients (up to 40%) (4, 5). Severe and recurrent chemotherapy−induced nausea and vomiting (CINV) may lead to dehydration, reduced nutritional status, electrolyte imbalance, metabolic disturbance, and weight loss, thereby significantly impairing patients’ quality of life and treatment tolerance. In extreme cases, CINV limits patients’ willingness to continue with chemotherapy, reduces treatment compliance, and may even lead to premature termination of therapy (6). Platinum−based doublet chemotherapy is widely used for NSCLC. Most platinum agents are classified as highly emetogenic chemotherapy drugs and often induce severe and prolonged CINV, especially delayed nausea and vomiting. Therefore, accurate assessment and timely preventive intervention are critical for patients receiving platinum−based chemotherapy (7).

In recent years, reducing the incidence and severity of CINV has become a key focus of clinical research. However, most studies primarily focus on pharmacological interventions, sometimes combined with traditional Chinese medicine, music therapy, or relaxation training (8). Insufficient attention has been paid to the underlying etiology, classification, severity grading, antiemetic efficacy evaluation, and the impact of CINV on patients’ daily function. Medical resources are not fully integrated, and standardized, individualized whole−process management strategies are still lacking.

Lung cancer chemotherapy is a complex procedure requiring multidisciplinary expertise, including oncologic treatment, nursing education, pharmacology, adverse reaction monitoring, nutritional support, and psychological intervention. Currently, multidisciplinary services are often fragmented across departments, leading to uncoordinated assessment and intervention and suboptimal CINV control. In addition, patients generally lack professional knowledge of CINV and tend to receive care passively rather than engaging in active self-management, which further reduces care effectiveness.

Multidisciplinary team (MDT) collaboration integrates professionals from different fields to optimize medical resources and provide patient−centered, individualized care. The core of MDT management is to optimize traditional workflows to meet personalized patient needs, reflecting the principles of voluntariness, interaction, integration, and cooperation (9). The MDT model has been widely adopted in oncology, critical care, respiratory medicine, neurosurgery, and other disciplines. Within this framework, nurses play a central coordinating role in continuous observation, symptom assessment, patient education, and care integration. A nurse−led MDT model effectively links multidisciplinary resources, enables comprehensive and timely evaluation, and facilitates patient−centered, whole−process care.

Chemotherapy is associated with multiple adverse reactions, among which CINV is particularly prevalent and troublesome. CINV is influenced by drug−related factors, chemotherapy regimens, antiemetic protocols, environmental factors, nutritional status, dietary habits, psychological conditions, and individual variability. It occurs across the entire chemotherapy trajectory: before, during, and after treatment. Routine care mainly focuses on episodic observation and drug intervention during chemotherapy administration, with limited assessment, brief monitoring, and insufficient understanding of CINV subtypes and risk stratification (10). Post−chemotherapy quality of life and long−term antiemetic efficacy across multiple cycles are rarely evaluated continuously. Nurses are key providers of symptom assessment, standardized drug administration, education, and whole−process management. Therefore, we designed a patient−centered, nurse−led MDT model for whole−process CINV management. This model integrates multidisciplinary resources to establish a standardized care pathway, reduce the incidence and severity of CINV, improve quality of life, and enhance the effectiveness of antiemetic care.

This study aimed to implement a nurse−led MDT model for whole−process management of CINV in patients with lung cancer undergoing chemotherapy. We observed the frequency and severity of CINV, explored the effect of the MDT model on CINV control, and clarified its value in preventing and managing CINV. We also sought to further standardize CINV care for patients with lung cancer.

The novelty of this study lies in three aspects: (1) constructing a nurse-led MDT model covering the whole process of chemotherapy (before, during, and after treatment); (2) implementing dynamic risk screening and personalized intervention for CINV; (3) integrating nursing, oncology, pharmacy, nutrition, psychology, and traditional Chinese medicine to form a standardized CINV management pathway. This study provides a practical and replicable strategy for CINV control in lung cancer patients.

## 2 Data and methods

### 2.1 Research objects

63 cases of non-small cell lung cancer (NSCLC) hospitalized in Cancer Center of Second Affiliated Hospital of Chongqing Medical University from July 2018 to December 2019 were selected as control, and routine nursing management was adopted. From January 2020 to June 2021, 63 patients with non-small cell lung cancer (NSCLC) hospitalized for chemotherapy were taken as experimental, and nurse led MDT management model was adopted for whole process management. The criteria for inclusion of patients were in line with definition criteria of non-small cell lung cancer in 2021 edition of Chinese Society of Clinical Oncology (CSCO) Guidelines for Diagnosis and Treatment of Non-small cell Lung Cancer.

#### 2.1.1 Inclusion criteria

Patients were included if they met the following criteria: (1) Pathological diagnosis of NSCLC confirmed by biopsy; (2) Adequate disease awareness; (3) Use platinum containing dual drug chemotherapy scheme; (4) Clear mind, normal ability to understand and express.

#### 2.1.2 Exclusion criteria

Patients were excluded if they: (1) Patients with consciousness disorder; (2) Suffered from nausea/vomiting caused by central nervous system metastasis or gastrointestinal obstruction; (3) Exhibited poor treatment compliance; (4) were diagnosed with mental illness and unable to cooperate with treatment.

#### 2.1.3 Chemotherapy administration

All subjects received systemic intravenous chemotherapy via central venous catheter access.

After diagnosis of non-small cell lung cancer is confirmed, responsible nurse and doctor in charge communicate diagnosis and treatment plan. According to treatment needs, a nurse with special operation technology qualification certificate of PICC in Chongqing will use Sadinger technology to conduct ultrasound guided percutaneous catheterization through peripheral vein (PICC), cooperate with doctor to complete placement of port device through upper arm, and use intracavitary ECG to assist positioning, The tip of central venous catheter is located in superior vena cava, providing a good channel for long-term intravenous treatment or chemotherapy.

#### 2.1.4 Sample size rationale

The sample size was calculated based on the expected incidence of grade ≥II CINV in the control group (approximately 60%) and a 20% reduction expected in the experimental group. With a statistical power of 80% (1−β=0.8) and a type I error α=0.05, the required sample size was approximately 60 patients per group. Finally, 63 patients were included in each group to compensate for possible dropout, totaling 126 cases.

### 2.2 Research methods and tools

The tools of this study include basic data questionnaire, antiemetic evaluation tool of Multinational Society for Cancer Support Therapy, Functional living index emesis (FLIE), and self-made questionnaire of patients’ satisfaction with management of nausea and vomiting caused by chemotherapy.

#### 2.2.1 Basic data questionnaire

A self-designed questionnaire was used to collect sociodemographic data of patients, including age, gender, weight and body mass index, tumor stage, history of alcohol allergy, history of motion sickness, education level, nutritional risk assessment, etc.; Collect general information of nurses, including age, gender, professional title, education background, working years, qualification of tumor specialist nurses, and knowledge of CINV and nauseated prevention management.

#### 2.2.2 Anti emetic evaluation tool of multinational association for cancer support therapy

CINV can be divided into acute vomiting (within 24h after chemotherapy) and delayed vomiting (24-120h after chemotherapy) according to occurrence time. Adverse reactions were observed according to CTI’s evaluation standard for common adverse events (CTCAE V4.0), and CINV was classified into 0-V grades. The scale includes 8 evaluation items in total.

(1) Nausea degree

Grade 0: no nausea;

Grade I: loss of appetite and unchanged eating habits;

Grade II: The intake of water decreases after oral intake, and intravenous rehydration is required, without weight loss, dehydration or malnutrition;

Grade III: There is obviously insufficient water for oral feeding, and continuous intravenous rehydration, nasal feeding or TPN are required;

Grade IV: serious complications, which may endanger life;

Grade V: death.

(2) Vomiting degree

Grade 0: no vomiting;

Grade I: vomit once within 24h;

Grade II: vomit 2 to 5 times within 24 hours, requiring intravenous rehydration;

Grade III: vomiting for more than 6 times in 24 hours, requiring intravenous fluid infusion, nasal feeding or TPN;

Grade IV: serious complications, which may endanger life;

Grade V: death.

#### 2.2.3 Nutrition risk assessment

The nutrition risk screening form recommended by Clinical Nutrition Department of Second Affiliated Hospital of Chongqing Medical University and PG-SGA nutrition assessment form for inpatients were used in this study.

(1) Nutrition Risk Screening Form

Applicable objects: ≥ 18 years old, ≤ 90 years old, hospitalization time>1 day, no operation before 8:00 next day, and clear mind.

Disease score:

1. Acute attack or complication of chronic disease, chronic obstructive pulmonary disease, hip fracture, hemodialysis, general malignant tumor, liver cirrhosis (1 point);
2. Major abdominal surgery, blood malignancies, severe pneumonia, stroke (2 points);
3. APACHE-II score>10 in ICU patients, bone marrow transplantation, brain injury (3 points);
4. No disease above (0 point).

Impaired nutritional status score:

1. Anthropometry: if BMI<18.5 and general condition is poor (3 points), if BMI ≥ 18.5 (0 point);
2. Weight status: if there is weight loss in near future (1 month to 3 months), weight loss within 3 months is more than 5% (1 point), weight loss within 2 months is more than 5% (2 points), and weight loss within 1 month is more than 5% (3 points);
3. Feeding status: If food intake decreases within one week, it will decrease by 25% - 50% (1 point), 51% - 75% (2 points), and 76% - 100% (3 points).

Note: Take highest value of above three summary scores.

Age score: if age is ≥ 70 years old (1 point), otherwise it is (0 point).

Note: total score of clinical nutrition screening=disease score + nutritional status impairment score (the highest score of 3 sub items) +age score.

Scoring requirements: If score is ≥ 3, notify doctor to ask clinical nutrition department to consult for nutritional evaluation. If score is less than 3, screen again one week later.

(2) PG-SGA Nutrition Assessment Form for Inpatient

The table is divided into patient self-assessment and medical staff (clinical nutritionist) assessment. The patient part includes weight, food intake, symptoms, activities and body functions; The evaluation of medical staff (clinical nutritionist) includes disease and its relationship with nutritional requirements, metabolic requirements and physical examination. Grade A means good nutrition; Grade B: moderate or suspected malnutrition; Grade C is severe malnutrition.

#### 2.2.4 Functional living index emesis

The functional living index emesis (FLIE), a Likert 7-grade scoring method, has 2 dimensions and 9 items. The evaluation scope is quality of life of patients 5 days after chemotherapy to assess impact of CINV on quality of life of patients. Each question is divided into 1 to 7 points. 1 point means that they are unable to complete daily life, and 7 points means that they can complete daily life well. The total score is 18 to 126 points. The lower total score, greater influence of nausea and vomiting caused by chemotherapy on patients’ daily life function, and lower quality of life of patients; On contrary, higher total score, smaller impact on daily life of patients, and higher quality of life of patients.

#### 2.2.5 The nurse led MDT management team manages whole process of nausea and vomiting caused by chemotherapy for lung cancer patients

(1) CINV multidisciplinary team collaboration management team members

MDT team composition: one deputy director of department is team leader, and one head nurse is deputy team leader and coordinator; 3 clinical specialized nurses who have worked in oncology department for more than 5 years, obtained professional technical title of nurse or above, obtained certificate of national or municipal oncology specialized nurse, and received narrative training; The MDT team consists of 3 oncologists with professional and technical title of attending physician or above, 1 TCM specialist with professional and technical title of attending physician or above, 1 clinical pharmacist, 1 clinical nutrition therapist, and 1 psychological consultant. The clinical specialist nurses are link of all professional personnel in entire MDT team. According to structure of nursing staff in department, N3 level and above nurses in tumor center are selected. It is required to have solid professional knowledge of tumor nursing, rich clinical experience, strong communication and coordination ability, humanistic care and narrative communication ability, active research and certain scientific research ability.

(2) Full process management process of CINV multidisciplinary team cooperation

1) The responsible nurse evaluated basic data of patients admitted to hospital, and lung cancer chemotherapy patients who met inclusion criteria were referred to MDT team after communication between oncology specialist nurse and doctor in charge. The three oncology specialist nurses collected data according to unified standards. The process was as follows: ① Create patient CINV case management file, and enter patient’s basic information into EXCLE system. Include patient data into department’s patient symptom monitoring and early warning reporting platform (platform source: real world data management platform (RWDMP)), and inform patients of application purpose and operation method of platform in advance; ② CINV risk screening: use self-made CINV patient risk assessment form to systematically and comprehensively assess patient’s medical history, physical examination, nutritional status, history of nausea and vomiting, lifestyle, education level, economic status, family support, self-management knowledge and behavior, Internet use, etc., and enter them into system to screen patients with different risk levels of nausea and vomiting, and choose different ways to intervene; ③ The MDT team conducts a comprehensive evaluation on chemotherapy scheme, antiemetic intervention, nutritional support, humanistic care, psychological counseling, etc. for patients; ④ Actively carry out personalized and diversified education and guidance on nausea and vomiting related knowledge of patients caused by chemotherapy, fully communicate with patients, and set expected management goals, including two goals: key indicators (nausea, vomiting, gastrointestinal discomfort, etc.) and behavioral status (eating, sleeping, mental status, etc.); Formulate prevention management plan under MDT mode, including specific process, main measures and detailed methods, such as team members’ communication plan, patient self-management health education plan, anti-vomiting prevention management measures, healthy diet plan during chemotherapy, reasonable activity and rest plan, nursing humanistic care and psychological counseling plan, discharge follow-up plan, etc.; ⑤ The incidence of nausea and vomiting caused by chemotherapy, life function index, patient experience and satisfaction were evaluated and recorded.

2) The MDT team led, implemented and tracked according to case management plan. The management cycle was 6–8 chemotherapy cycles.

① Leading plan: attending doctor shall formulate a treatment plan for anti-vomiting and anti-vomiting based on patient’s medical history and basic data, and deputy director of department shall be responsible for quality control and guidance of treatment plan; The clinical nurse is responsible for implementation of MDT links, health guidance and data recording, and head nurse is responsible for whole process monitoring and quality supervision; ② Multidimensional implementation of each chemotherapy cycle: clinical nurse implemented bedside CINV Prevention Intervention Inspection Record once, completed scientific popularization of “chemotherapy related nausea and vomiting health knowledge” on mobile phone once, bedside guidance of CINV Self-Management Health Manual, held two “non nausea management” health lectures every month, designed and completed “CINV awareness and prevention” bulletin board in ward, established a non-nausea management atmosphere, and improved patient participation and understanding; Nutritional therapists formulate reference recipes during chemotherapy, advocate use of two meal diet with high emetic, and do a good job of diet intervention and nutritional support; Clinicians comprehensively evaluated effects and side effects of chemotherapy scheme, and effects of antiemetic and antiemetic; Pharmacists provide professional medication guidance for refractory nausea and vomiting; In case of psychogenic nausea and vomiting or expectant nausea and vomiting, clinical nurses who have received professional training in narrative nursing should intervene and guide, and psychological consultants should intervene and guide when necessary to carry out multi-dimensional management of CINV details; ③ Follow up: before discharge, clinical nurse discussed with patient to determine follow-up method and time, and routinely selected fifth day after end of chemotherapy cycle 1 to carry out follow-up. Communicate and coordinate with doctors and nurses in a timely manner, and actively conduct remote processing or guide nearby medical treatment for patients who can use smart phones to receive nausea and vomiting related self-assessment symptoms from patients via RWDMP at any time; For patients who cannot use smart phones, follow up on 5th day after end of chemotherapy cycle 1 according to Discharge Follow up List of CINV Patients. A total of 2 chemotherapy cycles were followed up. The follow-up focuses on self-management behavior to maintain healthy eating habits, weight and good physical and mental status, occurrence of delayed nausea and vomiting and its impact on life function, evaluation of implementation of patient’s plan and achievement of set goals during next cycle of chemotherapy, and appropriate adjustment of target plan and intervention plan in combination with actual situation, providing personalized guidance to help patient achieve expected goals and promote effective coordination among team members, Achieve goal of MDT mode prevention and intervention. If nausea and vomiting symptoms are not improved, continue to carry out continuous and intensive intervention and management, and give reminders of future visits; ④ Stage evaluation: stage evaluation shall be carried out in one chemotherapy cycle, discussion within MDT, dynamic feedback of non-emetic control state, and timely adjustment of team intervention plan; ⑤ Mobile medical support: through portable mobile medical platform, real-time attention is paid to patient’s self-monitoring of nausea and vomiting, weight data, emotional and psychological status and other information, so as to timely discover patient’s problems. In case of high risk early warning, active care is taken to pacify patient, timely communication is made, and emergency treatment is coordinated.

### 2.3 Quality control

#### 2.3.1 Select bias control

**Figure 1:**
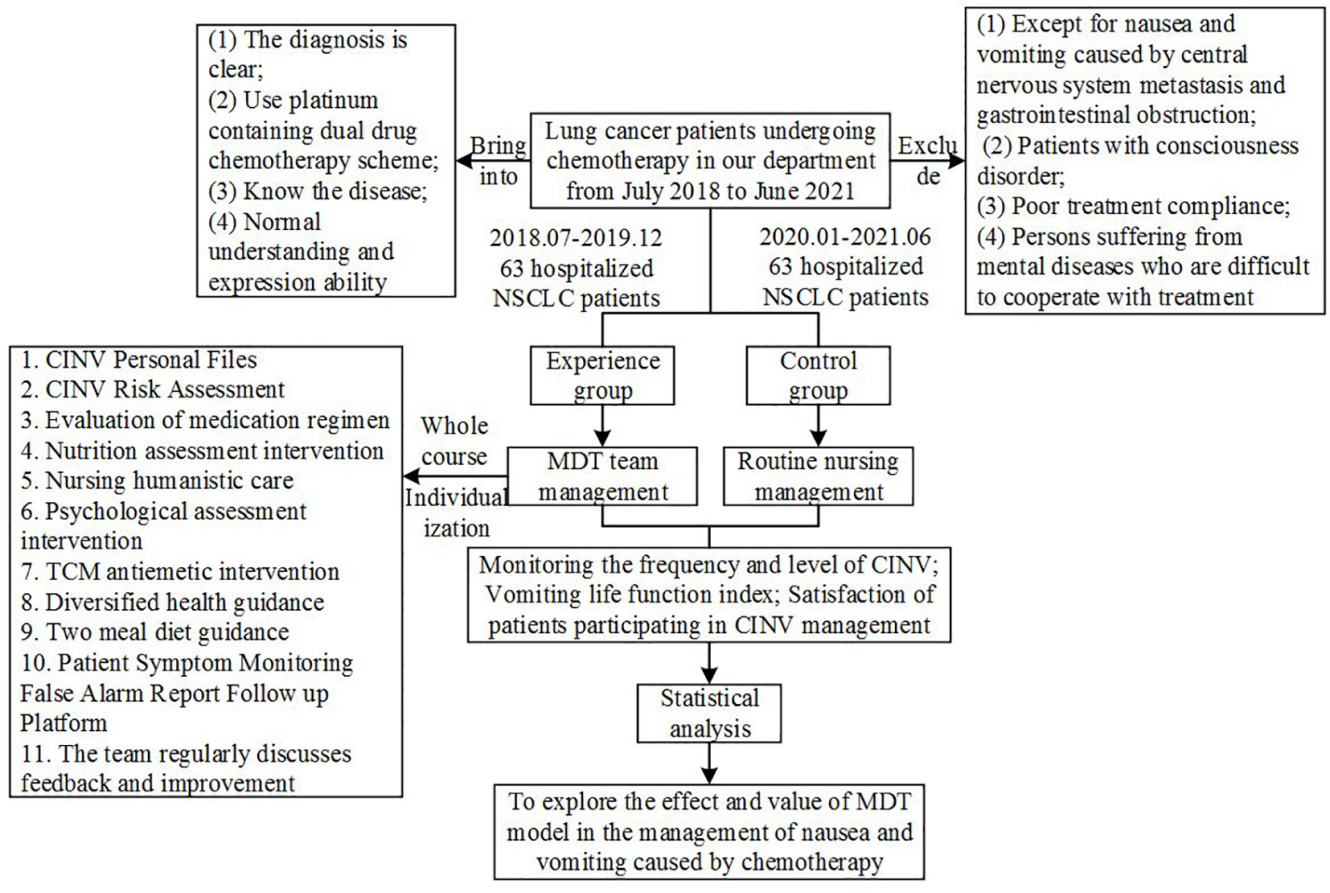
Treatment chart of lung cancer patients.

**Figure 2:**
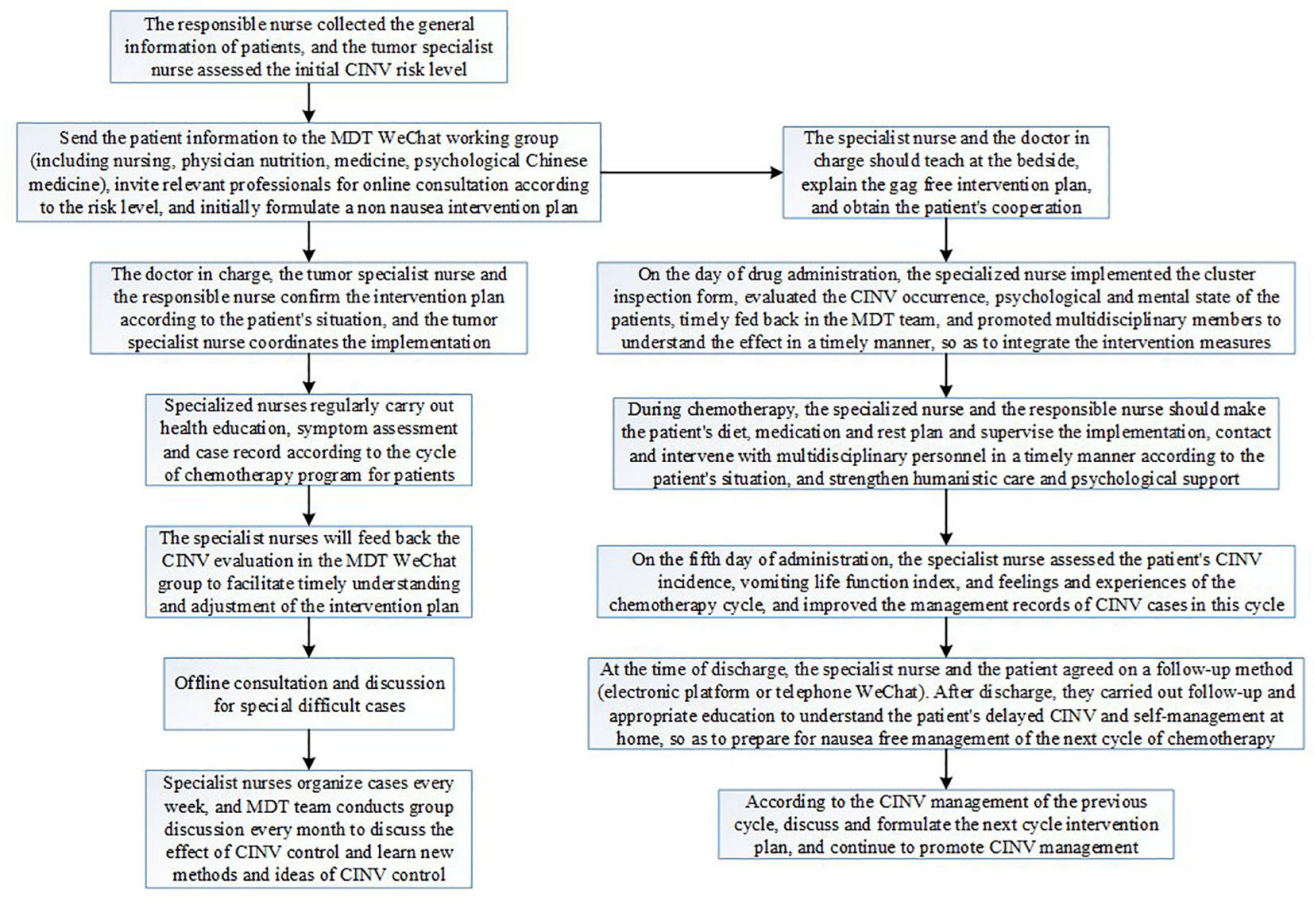
MDT intervention flow chart for nausea and vomiting caused by chemotherapy.

The subjects were selected in strict accordance with inclusion and exclusion criteria, and all patients who met inclusion and exclusion criteria during observation period were included to avoid selection bias. Technical route, see **Figure 1**. Flow chart of MDT intervention on nausea and vomiting caused by chemotherapy. See **Figure 2**.

#### 2.3.2 Measurement bias control

(1) The two clinical specialized nurses in MDT team uniformly used Shanghai Expert Consensus on Whole Process Management of Nausea and Vomiting Caused by Chemotherapy (2018 Edition) to assess nausea and vomiting, Nutrition Risk Screening Form and PG-SGA Nutrition Evaluation Form for Inpatients formulated by Clinical Nutrition Department of Second Affiliated Hospital of Chongqing Medical University to screen nutrition risk, and Vomiting Life Function Index Quantity Table to assess impact of patients’ quality of life, Patients who can use smart phones adopt electronic follow-up platform for self-monitoring management. Some patients who do not use smart phones, education and understanding will use CINV Patient Discharge Follow up Checklist for telephone follow-up, which will be recorded in CINV case management file;

(2) The general data of patients, such as vital signs, education level, weight, etc., were completed in Cancer Center Nurse Station of Second Affiliated Hospital of Chongqing Medical University using special measuring equipment (Omron upper arm cuff sphygmomanometer, Yuyue finger pulse oxygen monitor, weight scale);

(3) Chemotherapy administration route: all patients used central venous catheter (peripherally inserted central venous catheter, PICC; completely inserted venous infusion port, PORT) for systemic intravenous chemotherapy;

(4) All data shall be entered by two persons after being approved to avoid abnormal results.

### 2.4 Primary and secondary outcomes

The primary outcome was the incidence of clinically significant chemotherapy-induced nausea and vomiting (CINV), defined as grade ≥ II according to CTCAE v4.0, assessed on day 5 of chemotherapy cycle 2. Secondary outcomes included: (1) Quality of life assessed using the Functional Living Index-Emesis (FLIE) at day 5 after chemotherapy cycle 2; (2) Patients’ cognition and satisfaction regarding CINV prevention and management, measured using a self-designed questionnaire. Additionally, nurses’ cognition and mastery of CINV-related knowledge before and after MDT intervention were evaluated as exploratory outcomes.

### 2.5 Statistical analysis

All data were analyzed using SPSS 26.0 software. Enumeration data were expressed as case (percentage) and compared using the Chi-square test. Measurement data with non-normal distribution were expressed as median [P25, P75] and compared using the Mann-Whitney U test (between groups) or Wilcoxon signed-rank test (before-after intervention). A P-value <0.05 was considered statistically significant.

## 3 Results

### 3.1 Sociodemographic data of research object

There were 126 chemotherapy patients who met criteria of NEX. There was no difference in age, sex, body mass index, history of motion sickness, education level, nutritional risk assessment, tumor stage and other general data. See **Table 1**.

**Table 1:** Comparison of general data of patients.

Project name	Control (n=63)	Test (n=63)	Value	P value
Gender, n (%)	–	–	0	1
Male	39(61.90)	39(61.90)	–	–
Female	24(38.10)	24(38.10)	–	–
Age, media [P25, P75]	59[55, 66]	60[53, 66]	-0.535	0.593
Tumor staging	–	–	2.374	0.498
1	4(6.35)	8(12.70)	–	–
2	26(41.27)	27(42.86)	–	–
3	23(36.51)	22(34.92)	–	–
4	10(15.87)	6(9.52)	–	–
Body mass index, media [P25, P75]	21.13[19.48, 23.93]	21.09[19.03, 23.04]	0.656	0.512
Corona history, n (%)	13(20.63)	9(14.29)	0.881	0.348
Vomiting during pregnancy, n (%)	–	–	0.223	0.895
Not involved	39(61.90)	40(63.49)	–	–
Nothing	11(17.46)	12(19.05)	–	–
Have	13(20.63)	11(17.46)	–	–
Education level, n (%)	–	–	1.603	0.449
Primary school and below	16(25.40)	19(30.16)	–	–
Junior high school/senior high school	39(61.90)	40(63.49)	–	–
College degree or above	8(12.70)	4(6.35)	–	–
Nutrition risk score, media [P25, P75]	3[2, 4]	3[2, 4]	-0.114	0.909

Control, routine nursing management; Experimental, nurse-led MDT management.

### 3.2 Comparison of nurses’ general information and cognition of CINV before and after MDT intervention

**Figure 3:**
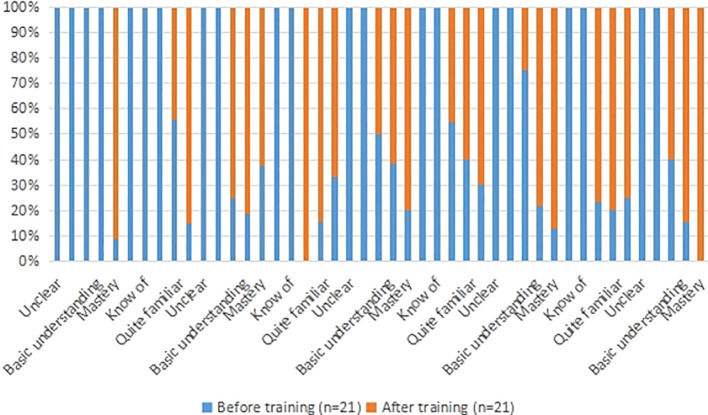
Comparison of nurses’ cognitive mastery of CINV before and after MDT intervention. Responses were categorized into four levels: Unclear, Basic understanding, Mastery, and Quite familiar. The blue bars represent the distribution before training, and the orange bars represent the distribution after training (n=21).

The nurses involved in this study were all staff members of Cancer Center of Second Affiliated Hospital of Chongqing Medical University and had qualification certificate of People’s Republic of China for nurses. A total of 21 nurses were involved. See **Table 2** for basic information. See **Table 3** and **Figure 3** for comparison of nurses’ cognition of CINV before and after MDT intervention.

**Table 2:** General information of nurses.

Category	-	-	Value (n=21)
Gender, n (%)	**-**	**-**	**-**
Male	**-**	**-**	1(4.8)
Female	**-**	**-**	20(95.2)
Age (years), media [P25, P75]	**-**	**-**	30[27, 33]
Education background, n (%)	**-**	**-**	**-**
Junior college	**-**	**-**	6(28.6)
Undergraduate	**-**	**-**	14(71.4)
Professional title, n (%)	**-**	**-**	**-**
Nurse	**-**	**-**	7(33.3)
Nurse	**-**	**-**	11(52.4)
Nurse in charge	**-**	**-**	3(14.3)
Years of service, media [P25, P75]	**-**	**-**	7[3,10]
Specialized nurse of oncology department, n (%)	**-**	**-**	**-**
Yes	**-**	**-**	3(14.3)
No	**-**	**-**	18(58.7)

**Table 3:** Comparison of nurses’ cognitive mastery of CINV before and after MDT intervention.

Project name	Before training (n=21)	After training (n=21)	P value
CINV Definition	–	–	<0.001
Unclear	7	0	–
Know of	4	0	–
Basic understanding	6	0	–
Quite familiar	2	0	–
Mastery	2	21	–
CINV Classification	–	–	<0.001
Unclear	6	0	–
Know of	3	0	–
Basic understanding	4	0	–
Quite familiar	5	4	–
Mastery	3	17	–
CINV classification	–	–	<0.001
Unclear	11	0	–
Know of	3	0	–
Basic understanding	1	3	–
Quite familiar	3	13	–
Mastery	3	5	–
Risk of drug induced vomiting	–	–	<0.001
Unclear	10	0	–
Know of	7	0	–
Basic understanding	0	6	–
Quite familiar	2	11	–
Mastery	2	4	–
More than 3 kinds of common intervention drugs are known	–	–	0.007
Unclear	5	0	–
Know of	4	0	–
Basic understanding	5	5	–
Quite familiar	5	8	–
Mastery	2	8	–
Know more than 3 common intervention methods	–	–	0.084
Unclear	2	0	–
Know of	4	0	–
Basic understanding	6	5	–
Quite familiar	6	9	–
Mastery	3	7	–
Cognition and attitude towards CINV	–	–	<0.001
Unclear	3	0	–
Know of	5	0	–
Basic understanding	9	3	–
Quite familiar	3	11	–
Mastery	1	7	–
Actively participate in CINV management	–	–	<0.001
Unclear	7	0	–
Know of	8	0	–
Basic understanding	3	10	–
Quite familiar	2	8	–
Mastery	1	3	–
Understanding of MDT management	–	–	<0.001
Unclear	8	0	–
Know of	7	0	–
Basic understanding	4	6	–
Quite familiar	2	11	–
Mastery	0	4	–

### 3.3 Comparison of CINV incidence and nausea and vomiting life index

The patients in control and experimental were evaluated for nausea and vomiting with Anti vomiting Evaluation Tool of Multinational Society for Cancer Support Therapy on day of administration of chemotherapy cycle 1, day of administration of chemotherapy cycle 5, day of administration of chemotherapy cycle 2, and day of administration of chemotherapy cycle 5. The evaluation results at each time point were compared and analyzed. The results showed that there were differences in frequency and severity of nausea and vomiting between test and control on day of administration of chemotherapy cycle 2 and fifth day of administration (P<0.05).

**Figure 4:**
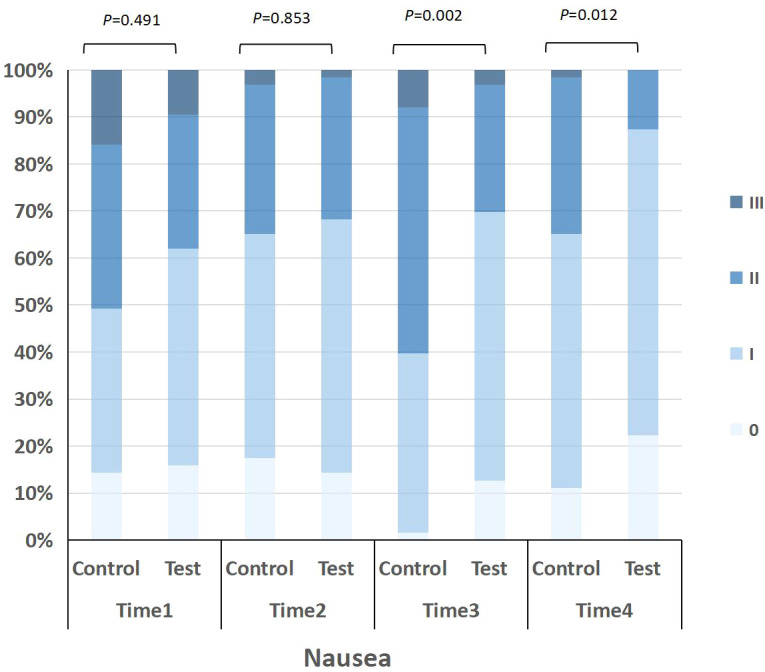
Distribution of nausea severity in the control and test groups at four time points (Time 1 to Time 4). Severity grades were defined as 0 (no nausea), I (mild), II (moderate), and III (severe). The stacked bar chart shows the proportion of patients at each grade, with p-values indicating between-group comparisons at each time point.

**Figure 5:**
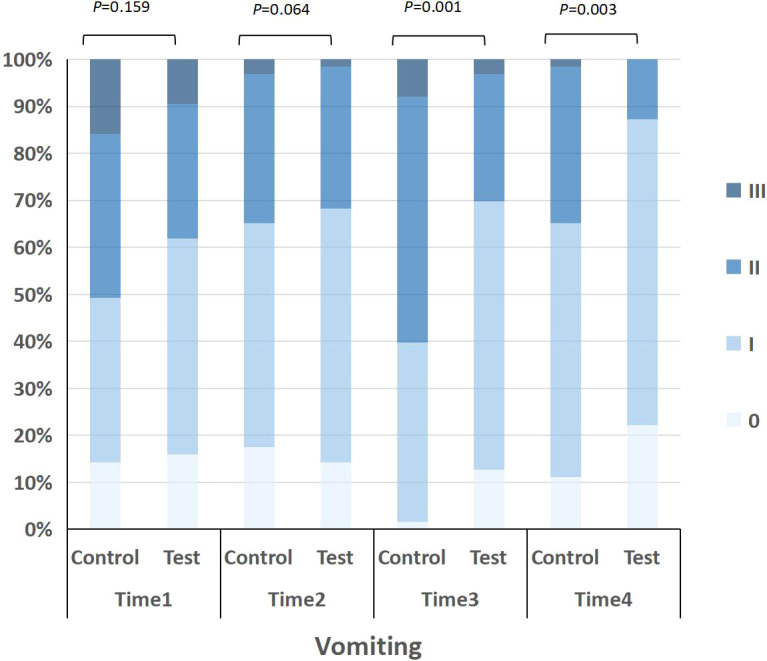
Distribution of vomiting severity in the control and test groups at four time points (Time 1 to Time 4). Severity grades were defined as 0 (no vomiting), I (mild), II (moderate), and III (severe). The stacked bar chart shows the proportion of patients at each grade, with p-values indicating between-group comparisons at each time point.

On fifth day after end of chemotherapy cycle 2, quality of life of patients was evaluated with Scale of Vomiting Life Function Indexes. See **Table 4**, **Figure 4** and **Figure 5** for details.

**Table 4:** CINV incidence and nausea and vomiting life index were compared.

Project name	Chemotherapy cycle and evaluation time points	Control (n=63)	Test (n=63)	Value	P value
Nausea index, media [P25, P75]		36[31, 39]	51[47, 53]	-9.548	<0.001
Chemotherapy cycle 1	0	9(14.29%)	10(15.87%)	–	0.491
Day of administration	I	22(34.92%)	29(46.03%)	–	
Chemotherapy cycle 1	II	22(34.92%)	18(28.57%)	–	
Day 5 of administration	III	10(15.87%)	6(9.52%)	–	
Chemotherapy cycle 2	0	11(17.46%)	9(14.29%)	–	0.853
Day of administration	I	30(47.62%)	34(53.97%)	–	
Chemotherapy cycle 2	II	20(31.75%)	19(30.16%)	–	
	III	2(3.17%)	1(1.59%)	–	
Chemotherapy cycle 1	0	1(1.59%)	8(12.70%)	–	0.002
Day of administration	I	24(38.10%)	36(57.14%)	–	
Chemotherapy cycle 1	II	33(52.38%)	17(26.98%)	–	
Day 5 of administration	III	5(7.94%)	2(3.17%)	–	
Chemotherapy cycle 2	0	7(11.11%)	14(22.22%)	–	0.012
	I	34(53.97%)	41(65.08%)	–	
	II	21(33.33%)	8(12.70%)	–	
	III	1(1.59%)	0(0.00%)	–	
Vomiting index, media [P25, P75]		34[30, 37]	51[46, 54]	-9.628	<0.001
Chemotherapy cycle 1	0	17(26.98%)	20(31.75%)	–	0.159
Day of administration	I	24(38.10%)	32(50.79%)	–	
Chemotherapy cycle 1	II	18(28.57%)	10(15.87%)	–	
Day 5 of administration	III	4(6.35%)	1(1.59%)	–	
Chemotherapy cycle 2	0	24(38.10%)	36(57.14%)	–	0.064
Day of administration	I	26(41.27%)	22(34.92%)	–	
Chemotherapy cycle 2	II	12(19.05%)	5(7.94%)	–	
	III	1(1.59%)	0(0.00%)	–	
Chemotherapy cycle 1	0	10(15.87%)	28(44.44%)	–	0.001
Day of administration	I	35(55.56%)	27(42.86%)	–	
Chemotherapy cycle 1	II	18(28.57%)	8(12.70%)	–	
Day 5 of administration	III	0(0.00%)	0(0.00%)	–	
Chemotherapy cycle 2	0	24(38.10%)	40(63.49%)	–	0.003
	I	31(49.21%)	22(34.92%)	–	
	II	8(12.70%)	1(1.59%)	–	
	III	0(0.00%)	0(0.00%)	–	

Control, routine nursing management; Experimental, nurse-led MDT management; CINV, chemotherapy-induced nausea and vomiting; FLIE, Functional Living Index-Emesis.

### 3.4 Comparison of patients on CINV prevention and control and satisfaction

On fifth day after end of chemotherapy cycle 2, self-designed Cognition and Satisfaction Questionnaire on CINV Prevention Management was used to investigate cognition and understanding of patients on CINV prevention management related knowledge and their satisfaction with CINV control. The results showed that after intervention, patients in test improved their cognition and satisfaction with CINV prevention management. See **Table 5**.

**Table 5:** Comparison of cognition and satisfaction of two groups of patients on CINV management.

Project name		Control (n=63)	Test (n=63)	Value	P value
CINV prevention awareness, n (%)		–	–	46.490	<0.001
Unclear		23(36.51%)	1(1.59%)	–	–
Partial understanding		34(53.97%)	23(36.51%)	–	–
Quite familiar		6(9.52%)	39(61.90%)	–	–
CINV control satisfaction		76[72, 80]	89[87, 92]	-9.383	<0.001

### 3.5 Adverse reactions

The patients received regular follow-up after chemotherapy for 3 months. No severe electrolyte disorder, anaphylaxis, neurotoxicity, life-threatening consequences, death and other adverse reactions occurred, but severe bone marrow suppression (Wbc<1.0) caused by chemotherapy occurred in individual cases, including 3 cases in control and 5 cases in experimental. There was no difference in occurrence of adverse reactions. The patients with adverse reactions were alleviated by active symptomatic treatment.

## 4 Discussion

Chemotherapy plays an important role in current comprehensive treatment of tumors and is one of main means of clinical treatment of tumors. In clinical practice, various anti-tumor treatments can cause nausea and vomiting, among which chemotherapy is most common and serious. Other drug treatments (molecular targeted drugs and pain relievers, etc.), radiotherapy and surgery may cause nausea and vomiting (11).

Chemotherapy−induced nausea and vomiting (CINV) is one of the most distressing treatment−related adverse reactions. Severe CINV leads to a series of gastrointestinal and systemic complications, reduces treatment tolerance, and may result in chemotherapy dose reduction, delay, or even premature termination. Although 5−hydroxytryptamine 3 (5−HT3) and neurokinin−1 (NK−1) receptor antagonists have improved antiemetic efficacy, more than half of patients still suffer from anorexia, malnutrition, weight loss, and physical and psychological distress. CINV severely impairs quality of life, increases fear of treatment, and is a major cause of nonadherence to effective chemotherapy (12).

CINV is classified into three types: acute, delayed, and anticipatory. Acute CINV typically occurs within 24 hours after the initial chemotherapy administration. Delayed CINV occurs 24 hours to several days after chemotherapy, which is commonly observed following platinum-based chemotherapy and may persist for multiple days (7).; Anticipatory Nausea and Vomiting (ANV) is a special type of nausea and vomiting caused by chemotherapy. Patients have experienced more than two cycles of chemotherapy, and nausea and vomiting begin before next use of chemotherapy drugs. The feature of ANV is that it can be induced by some factors related to chemotherapy, such as environment, smell, infusion apparatus, or mental factors such as insufficient antiemetic dosage or poor antiemetic effect during previous chemotherapy, anxiety and worry of patients (13). According to the Common Terminology Criteria for Adverse Events (CTCAE) version 4.0, nausea is graded based on oral intake and nutritional status, and vomiting is graded by frequency and clinical intervention requirements.

The occurrence of CINV is related to a variety of drug factors. The chemotherapy regimen, chemotherapy cycle, drug properties and vomiting are important factors for occurrence of CINV. The international guidelines classify chemotherapy drugs into four levels of emesis, namely, high risk>90%, medium risk 30%~90%, low risk 10%~30% and slight risk<10% (14). Platinum agents are highly emetogenic and frequently induce delayed CINV that is relatively resistant to antiemetic drugs (15). The risk of acute and delayed CINV is highest during the first two cycles and gradually decreases in subsequent cycles. In contrast, the risk of anticipatory CINV increases with more cycles. Patients with insufficient control during the first cycle are more than 3 times more likely to experience anticipatory nausea before their second cycle, supporting the need for individualized interventions (16).

Patient−related factors also strongly affect CINV, including age, sex, anxiety, motion sickness, and pregnancy−related vomiting. Female patients and those younger than 50 years have a higher risk of CINV, possibly related to hormonal differences (3, 17). Anxiety further increases susceptibility to CINV by enhancing conditioned reflexes and autonomic reactivity (18, 19). Interestingly, patients without a history of alcohol consumption have a higher CINV risk, which may be related to differences in drug metabolism and neurotransmitter sensitivity (20). Environmental factors in the ward, such as smells, sounds, and visual cues, also contribute to CINV.

Nurses play a central role in continuous CINV assessment, education, and whole−process management. Subjective patient−reported tools are widely used to evaluate CINV, including the MASCC Antiemesis Tool (MAT), the Index of Nausea, Vomiting, and Retching (INVR), the Morrow Assessment of Nausea and Emesis (MANE), and the Functional Living Index−Emesis (FLIE). The FLIE questionnaire specifically measures the impact of CINV on daily activities and quality of life.

For non−small cell lung cancer, platinum−based doublet chemotherapy is standard. Guidelines recommend triple antiemetic prophylaxis including a 5−HT3 antagonist, dexamethasone, and an NK−1 antagonist for highly emetogenic regimens. Adjuvant strategies include acupuncture, dietary interventions, frequent small meals, psychological support, relaxation training, and narrative nursing.

Despite advances in antiemetic therapy, CINV management remains fragmented due to insufficient multidisciplinary collaboration. The complexity of chemotherapy requires integrated expertise from oncology, nursing, pharmacy, nutrition, psychology, and traditional Chinese medicine. The multidisciplinary team (MDT) model optimizes resource integration and provides patient−centered, coordinated care. Nurses are uniquely positioned to lead MDT efforts given their continuous presence and role in education, coordination, and long−term follow−up.

A nurse−led MDT model enables whole−process CINV management spanning pre−chemotherapy risk assessment, intra−treatment monitoring, and post−discharge follow−up. This approach improves early intervention, standardized education, personalized diet, psychological support, and timely antiemetic adjustment. By unifying care pathways, the model enhances nurses’ knowledge and competence in CINV management and strengthens patient participation in self−care.

The present study demonstrates that a nurse−led MDT model significantly reduces the frequency and severity of CINV, especially during the second chemotherapy cycle. Patients in the MDT group had higher FLIE scores, better understanding of CINV, and greater satisfaction with care. These findings confirm that structured multidisciplinary care improves symptom control, treatment tolerance, and quality of life.

Nevertheless, this study has limitations. First, it was a retrospective, non−randomized historical control study, which may introduce temporal bias. Second, the sample was limited to non−small cell lung cancer, limiting generalizability. Third, the intervention period was relatively short. Future prospective randomized controlled trials with larger samples and broader tumor types are warranted to validate the nurse−led MDT model and support the establishment of standardized antiemetic care wards.

## Data Availability

The original contributions presented in the study are included in the article/supplementary material. Further inquiries can be directed to the corresponding author.
